# The Incidence of *Clostridioides difficile* Infection in the Post-COVID-19 Era in a Hospital in Northern Greece

**DOI:** 10.3390/diseases12080190

**Published:** 2024-08-20

**Authors:** Maria Terzaki, Dimitrios Kouroupis, Charalampos Zarras, Dimitrios Molyvas, Chrysi Michailidou, Panagiotis Pateinakis, Konstantina Mpani, Prodromos Soukiouroglou, Eleftheria Paida, Elisavet Simoulidou, Sofia Chatzimichailidou, Konstantinos Petidis, Athina Pyrpasopoulou

**Affiliations:** 1 2nd Propedeutic Department of Internal Medicine, Hippokration General Hospital Thessaloniki, 54642 Thessaloniki, Greece; mary.terz@hotmail.com (M.T.); dimcour841@gmail.com (D.K.); dmolyvas@gmail.com (D.M.); pateinakis@hotmail.com (P.P.); i.paida@windowslive.com (E.P.); elisavetsim@gmail.com (E.S.); sofia_hatzi75@hotmail.com (S.C.); petidisk@med.auth.gr (K.P.); 2 Microbiology Laboratory, Hippokration General Hospital Thessaloniki, 54642 Thessaloniki, Greece; zarraschak6@gmail.com (C.Z.); chrysimicha_beni@yahoo.gr (C.M.); nantiabani@hotmail.com (K.M.); maksouko@yahoo.gr (P.S.)

**Keywords:** *Clostridioides difficile*, incidence, nosocomial, surveillance

## Abstract

*Clostridioides difficile* infection (CDI) has evolved to be the most significant cause of healthcare-associated diarrhoea and one of the leading representatives of healthcare-associated infections, with a high associated mortality. The aim of this retrospective study was to record the incidence rates and the epidemiological and clinical features of CDI in a large tertiary hospital of northern Greece in the years 2022-2023. All patients with CDI-compatible symptomatology and a positive CDI diagnostic test (GDH—glutamate dehydrogenase and toxin-positive FIA—Fluorescent Immuno-chromatography—SD Biosensor, and/or film array) were included (104 from a total of 4560 admitted patients). Their demographic, laboratory, and clinical data were recorded and analysed. The incidence of CDI in admitted patients was found to be higher than previous reports in the geographical area, reaching 54.6/10,000 patient days and following a rising trend over the course of the study. Thirty-day mortality was high (39.4%), potentially related to new emerging hypervirulent *C. difficile* strains. In view of the high prevalence of multidrug-resistant organisms in the region, and the significant mortality associated with this infection, these findings particularly point to the need for the implementation of organized surveillance and infection prevention protocols.

## 1. Introduction

*Clostridioides difficile* infection (CDI) ranks as a leading cause of healthcare-associated infections [1]. The implicated pathogen is a Gram-positive, anaerobic, spore-forming bacterium, known to have causal association with diarrhoeal infections and, recently, also with colon cancer [2]. The syndromic clinical spectrum may vary from mild diarrhoea to the development of pseudomembranous colitis and toxic megacolon. Its severe clinical forms are associated with significant morbidity and mortality [3]. Major predisposing factors to the development of CDI appear from observational epidemiological studies to be prior recent admission, advanced age (>65 years), and recent administration of antibiotics, mainly including, but not restricted to, clindamycin, fluoroquinolones, and third-generation cephalosporins [4,5].

The majority of patients develop healthcare-associated CDI ≥ 48 h into the course of their hospitalization [6]. Surveillance and infection prevention policies have therefore focused on minimizing its nosocomial spread. To a much lesser extent, variable among the different epidemiological reports but usually reaching at maximum one-third of the total cases, *C. difficile* colonization ± infection may be recorded in the absence of recent healthcare exposure. Although the implicated strains may not necessarily differ, the clinical course in community-acquired (CA) CDI may be more indolent. The origin of CA *C. difficile* may be traced to asymptomatic humans of usually younger age, animals, food sources, and the environment [7].

Prior to the SARS-CoV-2 pandemic, CDI incidence was reported, on average, in lower frequencies in European countries than in Northern America [8,9,10] or other developed countries [11]; surveillance reports, however, have not, to a significant extent, been very diligent. In the past decade, both in the US as well as in Europe, the incidence of HA-CDI appeared to be decreasing, while CA-CDI tended to increase [12,13].

The pandemic apparently had a variable impact on the epidemiological data of CDI among different countries. In countries like Germany, during this time period, a significant decrease in CDI has been recorded, which may have coincided with the preceding implementation of strict antibiotic stewardship policies, e.g., the reduction in fluoroquinolone consumption [14]. A recently published retrospective study from a large tertiary hospital in northern Greece reported increasing trends of CDI incidence among hospitalized patients prior to the pandemic (2018), with the increase being much more prominent during the pandemic [15]. The aim of this study was to record CDI prevalence among inpatients of a medical ward of a large tertiary hospital in northern Greece in the post-COVID-19 era. The epidemiological features of the patients were analysed and their association with clinical severity and outcome was tested statistically. Potential associations with other epidemiological features of the analysed time period are discussed.

## 2. Materials and Methods

Study design and setting: The Hippokration Hospital of Thessaloniki is the largest tertiary hospital of northern Greece, with 800 beds, admitting patients of all medical subspecialties, including immunosuppressed, haematological, oncological, and transplant patients, and featuring a large Adult Patient Intensive Care Unit with 30 beds. The hospital regularly admits patients from rehabilitation centres and chronic care facilities, and critically ill patients who were previously admitted to the Intensive Care Unit continue their treatment after extubation on regular wards.

Conducting the study and analysis:

A retrospective study on patients diagnosed with CDI infection on the medical wards of Hippokration Hospital in the post-COVID-19 era was performed to assess the incidence of the disease among inpatients and its evolution over time. Parameters such as demographics and patient-related epidemiological data were recorded and associated with clinical severity and outcome.

Inpatients of the largest hospital medical department from 1 January 2022 to 31 December 2023 with compatible clinical symptomatology (diarrhoea, abdominal pain, abdominal distension, vomiting, fever, leucocytosis, and/or increased inflammatory markers [16]) and a positive test result for CDI were included in the study. Faecal samples from patients with diarrhoea were screened with the GDH FIA (Fluorescent Immuno-chromatography—SD Biosensor) test and/or the film array gastrointestinal panel (Biomérieux, France). Patients were classified as positive for CDI if they had a positive immunoassay test for the CDI antigen (Ag) and one or both of the CDI toxins (A/B), and/or if they had a positive film array test for an enterotoxigenic *C. difficile* strain along with compatible clinical symptomatology and no detection of other potentially implicated pathogens [17]. A colonoscopy was not routinely performed.

The patients’ epidemiological features and comorbidities, the origin of infection [healthcare associated—(HA) and community acquired—(CA)], and recent SARS-CoV-2 infection and/or antibiotic use were recorded. The origin of infection was characterized as healthcare-associated either if it developed during admission (healthcare facility-onset (HO) CDI) or if it manifested in outpatients with recent healthcare facility exposure within 3 months prior to admission (community-onset, healthcare facility-associated (CO-HCFA) CDI). A previous history of CDI was noted (CDI recurrence within 8 weeks of the previous episode). The severity of infection was classified as mild/moderate, severe, or fulminant [18]. Intensive Care Unit admission and outcome (death within 30 days) were recorded. The severity of infection and the outcome were correlated with the detection of toxins, and the outcome was correlated with epidemiological parameters, the history of the patient (comorbidities and previous SARS-CoV-2 infection), the origin of the infection, and ICU admission. The total incidence of CDI was calculated in cases/10,000 patient days (pt days). Single-day admissions were excluded from the analysis. Statistical analysis was performed with SPSS 26.0. The normality of the distribution of continuous variables was tested by the one-sample KolmogorovSmirnov test. Associations between categorical variables were tested with the chi-square test, and associations between continuous and categorical variables were tested with the independent samples *t*-test. Statistical significance was set by *p* < 0.05. This study was approved by the Hippokration Hospital Ethics Committee (Approval No.: 17113/9-4-2024).

## 3. Results

From a total of 4560 admissions, from 1 January 2022 to 31 December 2023, 104 patients who presented with, or developed, CDI-relevant symptomatology had a positive faeces test for toxin-producing *C. difficile*. Among those, 42 were male (40.4%); the median age of the patients was 80.0 years [18–97]. A significant majority of the patients (80.8%) were admitted from home; 52.9% of the patients had, in the previous 4 months, been admitted to a healthcare facility. In 81 patients (77.9%), antibiotic use in the past trimester was recorded; among patients admitted from home, without recent hospitalization, the most commonly used antibiotics on an outpatient basis were quinolones, followed by cephalosporins and broad-spectrum b-lactams. SARS-CoV-2 infection within the previous year was recorded in 38 (36.5%) patients. The demographic and epidemiological data are summarized in Table 1.

Seventy-four (74) patients were diagnosed with a positive PCR for a toxin-producing CDI strain; thirty (30) were only tested using GDH and positive toxin(s) FIA. In 88 patients, the infection was considered to be healthcare- and healthcare facility-associated (Table 1). Sixty-five patients (62.5%) developed CDI during their admission (onset following admission), with a median duration of hospitalization prior to CDI development of 4.0 days; in 16 cases (15.4%), CDI was considered to be community-onset. In 8.3% of the patients, it was considered a recurrent episode of a previously recorded CDI infection in their medical history.

**Figure 1:**
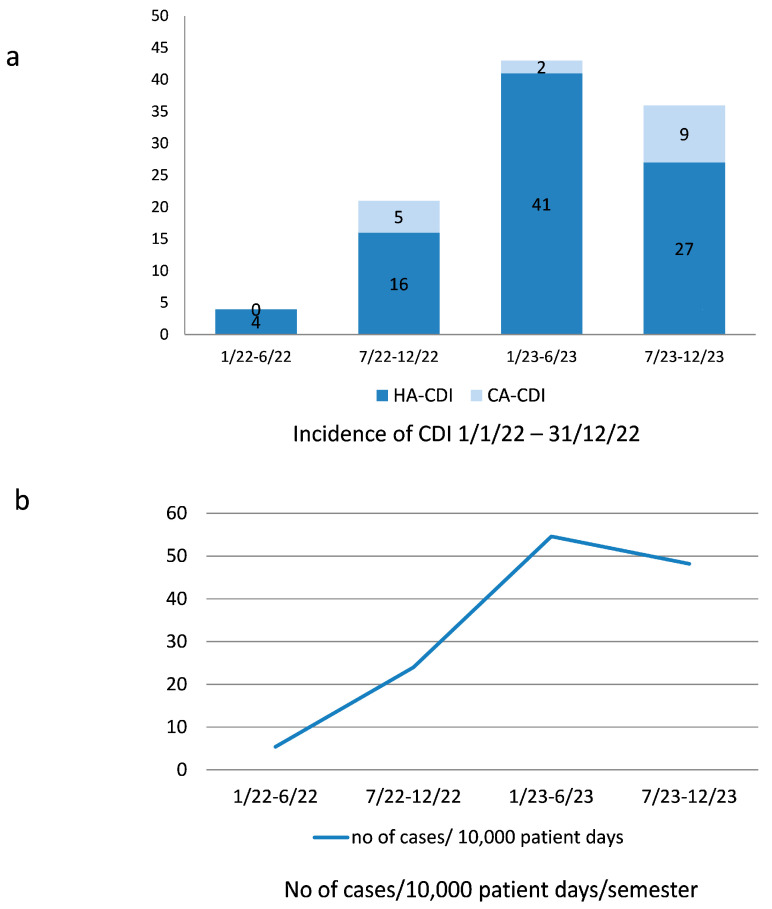
Up to 31 December 2023, incidence of CDI among inpatients of medical wards of Hippokration Hospital. (**a**) Total cases of community-acquired (CA) CDI and healthcare-associated (HA) CDI per semester. (**b**) CDI incidence in cases/10,000 patient days.

The patients were classified according to the severity criteria of the American College of Gastroenterology [19] as having mild/moderate (51.9%), severe (26.9%), or fulminant (21.2%) disease. Forty-one (39.4%) patients died within the first 30 days of diagnosis. Among those, in 13,4% of the total (14 patients), death was directly related to CDI infection. In the rest of the patients, death occurred mostly in the context of new-onset sepsis within the 30-day period. Thirty-day mortality was not associated with gender, age, the number of comorbidities, the length of hospitalization prior to a positive CDI test, the origin of infection, the semester of diagnosis, or previous COVID-19 infection (Table 2). It was associated with severity of infection and ICU admission (Table 2). CDI prevalence rose from 0.3% in the first half of 2022 to 1.8% in the second half, to 2.4% in the first half of 2023, and to 2.9% from 1 July 2023 to 31 December. When converted to incidence/10,000 patient days, the corresponding recorded incidence was 5.4, 24.0, 54.6, and 48.2 cases/10,000 patient days/semester in the time periods mentioned above, respectively (Figure 1a,b). The treatment of initial episodes usually involved oral/enteric administration of vancomycin; recurrent episodes were usually treated with fidaxomicin. Probiotics were not routinely administered during the acute infection.

## 4. Discussion

This is a retrospective epidemiological study on the changing incidence of *Clostridioides difficile* infection in a large tertiary hospital of northern Greece in the post-COVID-19 era (2022–2023); epidemiological and clinical features are analysed. *C. difficile* infection is a major cause of nosocomial morbidity and mortality. In European epidemiological reports from roughly a decade ago, the estimated burden of the disease annually appeared to involve about 130,000 patients, leading to more than 12,000 deaths [20]. Symptomatology may present within a wide range of severity, from mild diarrhoea to severe colitis, toxic megacolon, sepsis, and multiorgan failure [21]. The pathophysiology of the disease relies on the ability of the vegetative form of the anaerobic bacterium to produce toxins, pore-forming proteins causing gangrene and gastrointestinal complications [22]. They function by inactivating Rho-GTPases, regulators of the eukaryotic cytoskeleton, thus disrupting the tight junctions among intestinal epithelial cells and causing failure in intestinal barrier function [23]. The impacted damage of the intestinal epithelium in turn triggers the immune response and evokes clinical symptomatology. The expression of the two hallmark toxins (tcdA and tcdB) is essential for the development of the disease. In vitro experiments with genetically modified *C. difficile* strains on cell lines showed that either one of these toxins are cytotoxic, although the activity of toxin A is more pronounced than that of B [24]. Their expression is affected by various environmental conditions, such as the availability of specific nutrients, temperature, cell density, redox potential changes, phage infection, and the presence of antibiotics [25]. Since 1987, a third, previously under-recognised, binary toxin has been known to be produced by some *C. difficile* strains. This binary toxin is found in most non-toxinotype 0 strains [26]. Non-toxigenic strains may simply colonize the colon and protect the hosts from infestation by virulent, pathogenic strains. Indeed, non-toxin proteins have been studied for *C. difficile* vaccine development [27].

According to the 2017 Infectious Diseases Society of America (IDSA)/Society for Healthcare Epidemiology of America (SHEA) guidelines, testing for CDI is recommended for patients with unexplained new-onset diarrhoea with three or more unformed stools in 24 h [19]. A glutamate dehydrogenase (GDH, a protein produced by both toxin-producing and non-toxin-producing *C. difficile* strains)-positive FIA test is a cornerstone of diagnosis [21]. To firmly establish their presence, *Clostridioides difficile* toxins should also be tested and detected by FIA. A significant drawback is the quantity of the toxins required for a positive test, and the impact of sample mishandling on test result reliability. The film array (Nucleic Acid Amplification Testing—NAAT) is able to accurately identify toxigenic strains. Testing in our patients was prompted by clinical symptomatology (mainly unexplained diarrhoea, but also abdominal pain, distension, and increased inflammatory markers). Diagnosis was based on GDH- and toxin-positive (A/B) FIA and/or NAAT testing. Clinical severity was associated with ICU admission and death within 30 days, with similar findings to analogous reports in the literature [28].

Impressively, the incidence of CDI, according to our results, rose significantly within a period of 2 years (1 January 2022–31 December 2023) from 5.4 cases/10,000 patient days to 54.6 cases/10,000 patient days/semester, mainly involving healthcare-associated infections. Greece has historically been considered a country with a low incidence of CDI, with reports of incidence ranging from 3.7 to 5.6/10,000 patient bed days [10,29]. A low clinical suspicion and testing rate, as well as a lack of more sensitive tests for the detection of toxigenic strains, such as NAAT testing, may, to some extent, partially account for this observation. Recently, a retrospective study on the frequency of CDI in hospitalized patients in another tertiary hospital in northern Greece showed a significant increase from very low rates to >11 cases/10,000 patient days in the years prior to and during the pandemic (from January 2018 to March 2022) [15]. Unpublished data from our hospital from the year preceding our study (2021) revealed an estimated prevalence of 6.3 cases/10,000 pt days. This study shows a continuous, further increasing tendency in terms of incidence rates in the time period that followed. The association of CDI with antibiotic consumption is well established [30]; an explanation for this alarming increase in the incidence of the disease could stem from the COVID-19 pandemic and the extensive antibiotic prescription rate during this period. An association between previous COVID-19 infection and the development of the disease could not be established in our study.

Another pandemic-related explanation for this phenomenon may well be the constant ward rearrangements that occurred during this time period in Greek hospitals, impeding patient isolation; the extended use of other healthcare facilities, e.g., rehabilitation centres; and the dense, in terms of space occupation, hospitalization of inpatients. The transmission and horizontal spread of the pathogen is facilitated mainly by its ability to form endospores which are very resilient to high temperatures and disinfectants [31]. To prevent intranosocomial outbreaks, several methods can be implemented, including the use of chemical disinfectants, hydrogen peroxide vapour (HPV) systems, and ultraviolet (UV) radiation [32]. Impaired host response to bacterial implantation and new emerging strains with hypervirulent characteristics may also be of determining importance [33,34,35]. CDI is a significant cause of inpatient mortality. Forty-one of our CDI patients (39.4%) died within 30 days of diagnosis, in association with the severity of the disease. Although other pathological causes of death contributed, the length of stay was not associated with the outcome. Molecular typing of the implicated strains could not be performed as this was a retrospective study; thus, a direct correlation of strain type with clinical severity and manifestations is lacking.

In conclusion, CDI appears to be becoming an emerging threat even in countries not previously as severely affected. The increasing incidence of CDI in combination with the emergence of new, hypervirulent *C. difficile* strains [36] renders the implementation of surveillance programs, both in terms of the prompt recognition and reporting of CDI cases as well as the molecular characterization of the circulating strains, mandatory. In countries such as Greece which are in the lead of multidrug-resistant pathogen prevalence, strict antibiotic stewardship and restriction programs need to be implemented to halt further aggravation of the epidemiological situation and protect public health.

## Figures and Tables

**Table 1:** The epidemiological and clinical characteristics of the patients with CDI.

Parameter	
Gender, male: n (%)	42 (40.4)
Age (years) (median, range)	80.0 [18–97]
Admission from home (n, %)	84 (80.8)
Recent admission (n, %)	55 (52.9)
Recent treatment with Abs (n, %)	81 (77.9)
Healthcare associated (healthcare facility-onset (HO) CDI and community-onset, healthcare facility-associated (CO-HCFA) CDI) (n, %)	88 (84.6)
Recurring infection (n, %)	11 (10.6)
No. of comorbidities (median, range)	2.0 [0–4]
Immunosuppression (n, %)	31 (29.8)
SARS-CoV-2 infection in the previous year (n, %)	38 (36.5)
Duration of admission prior to positive CDI test (median, range)	4.0 [0–52]
Severity of infection (severe; n, %)	28 (26.9)
Severity of infection (fulminant; n, %)	22 (21.2)
ICU admission (n, %)	7 (6.7)
Death 30 days (n, %)	41 (39.4)

CDI, *Clostridium difficile* infection; No./n, number; ICU, Intensive Care Unit; Abs, antibiotics. Recorded comorbidities: cardiovascular disease, diabetes mellitus, chronic obstructive pulmonary disease, neurological/cerebrovascular disease, cirrhosis, malignancies, and immunosuppression.

**Table 2:** Association of CDI outcome (30 days) with epidemiological and clinical parameters.

Variable	Death	Recovery	*p*	95% CI
Age (years; mean)	76.61	74.97	0.588	−7.948–2.031
Gender (male; %)	41.5	39.7	0.856	NA
No. of comorbidities (mean)	1.46	1.70	0.224	−0.146–0.616
Days of hospitalization (days; mean)	7.59	5.84	0.283	−4.951–1.462
Healthcare-associated (yes; %)	90.2	77.8	0.118	NA
Previous COVID-19 infection (yes; %)	39.0	34.9	0.683	NA
Severity of infection (1—mild/moderate, 2—severe, 3—fulminant) (mean)	NA	NA	0.000	NA
ICU admission (yes; %)	17.1	0.0	0.001	NA

## Data Availability

The original contributions presented in this study are included in the article. Further inquiries can be directed to the corresponding author.
